# Concussion Injuries to the Visual Apparatus

**Published:** 1918-08

**Authors:** 


***An Address on Concussion Injuries of the Visual Apparatus in Warfare, of Central Origin.*** By S. A. Kinnier Wilson. *The Lancet,* No. 4897, Vol. CXCIII.

*Cerebral Concussion and Commotio* : In most cases of severe blows on the head which have proved fatal, examination has shown the presence of capillary hemorrhages, and consequent disintegration of myelin, such as are sufficient to account for the clinical symptoms.

In non-fatal cases it is justiciable to assume organic lesions such as have been enumerated, which have not, however, been sufficiently widespread or serious to affect permanent impairment of the cerebral function.

The question of molecular concussion or commotio presents from the clinical side features of importance. Clinically there is, at the one extreme, a total motor and sensory paraplegia ; at the other, the tremulous, weakness, paresthesia, and exaggerated reflectivity of " functional " disease. Experiments performed by Alan Newton suggest that there is an organic basis for the transient disturbances of function seen in so-called " railway spine " and allied conditions of traumatic neurasthenia, and they bear out what has adready been remarked that "functional" symptoms may be the expression of minimal organic changes. They also prove the occurrence of a relatively surprising amount of tissue change from a feeble impact on the exposed cord, and show that the blow capable of producing merely a molecular concussion must be slight.

While gross contusion lesions of the central visual apparatus are common enough one may have to seek far and wide ere one can find a case which for the purposes of this communication may be admitted into the category of visual concussion, where, one may suppose, the organic disturbances are of the trivial nature that has already been described.

After two and one-half years one is still looking for a case where a direct blow on the head from any form of projectile, without gross wounding, has resulted in an uncomplicated visual commotio analogous to the cases of spinal-card or peripheral-nerve commotio that have been, relatively speaking, common.

Let it be observed that all cases of visual disturbances from shell explosions *in the vicinity of* the individual concerned are purposely excluded.

All cases of head wounds with resultant and more or less permanent organic visual defects are also excluded.

We are left, then, with cases where a blow on the head produces visual symptoms that tend to clear up, but does not result in a permanent hemianopia in any of its varieties or other type of organic symptoms.

In a case mentioned by the author, as X-ray and the fact of operation indicate, we are dealing with a lesion of undoubted severity, the outer table being splintered and the inner being cracked, and perhaps slightly splintered also. It must be borne in mind that the patient was unconscious for at the most three minutes, that there was no evidence of intracranial pressure, that the absence of any form of scotoma or hemianopia was proof of the general integrity of the visual cortex, and that the vesico-psychic areas were similarly intact.

If, therefore, there is structural integrity of the visual cortex, we are justified in associating the visual phenomena with the fact of a violent concussion over the occipital poles. The following are the reasons for not regarding the case as traumatic hysteria:

With the exception of the visual symptoms, no impairment of function of the nervous system was discovered. Concentric diminution of the visual fields has never been described as an isolated phenomenon in hysteria. Moreover, an interval of hours or days intervenes between the shock-producing stimulus and the development of a traumatic psychoneurosis such as hysteria.

By way of contrast, cases may be selected in which, notwithstanding head injury and cerebral concussion amounting perhaps to contusion, the visual symptons are derived from another source. In some cases, in spite of the general cerebral concussion, unconsciousness, and immediate blindness, the persistant visual defect is clearly seen to be of the nature of so-called " subconscious malingering ".

The question of interpretation of the visual phenomena in the first case quoted is of considerable importance. The view here put forward is that symptoms usually held to be " functional " in type may be the expression of a direct visual concussion, undoubtedly an organic condition. These symptoms are concentric restriction of the visual fields and the occurrence of helicoid or spinal fields with certain tests.

In regard to the first of these, it must be borne in mind that such concentric diminution supposed to be characteristic of hysteria does certainly occur from head injuries.

In certain cases of organic injury of the central vision mechanism, by concussion or otherwise, the result is constriction of both fields, occurring by itself and without evidence of subconscious vision in the remainder.

The author has not been able to find any reference to helicoid fields in recent war literature with the exception of a comment by the late M. Jessop at the discussion on shell shock without visible signs of injury, at the Royal Society of Medicine. Jessop there stated that he had had under his care numerous cases of temporary blindness following the explosion of shells. " The blindness lasted under a week, and generally about two or three days. Both eyes were always affected, the pupils were active to light and the ophthalmoscopic appearances normal. The fields of vision were sometimes contracted, but more often normal; in only two cases did I find a spinal field.

For the present, it appears a reasonable contention that in certain cases without permanent visual defect of the accepted organic type of hemianopia or scotoma constriction of the fields, and helicoid or spinal fields, may be the expression of an organic change, the basis of which is a violent commotio, or a concussion amounting to contusion, of the visual cortex or some part or parts of it not more closely to be specified, or of the subcortical visual projection system.

